# Storage stability and *in vitro* digestion of apigenin encapsulated in Pickering emulsions stabilized by whey protein isolate–chitosan complexes

**DOI:** 10.3389/fnut.2022.997706

**Published:** 2022-09-29

**Authors:** Ruihong Ge, Haihua Zhu, Jian Zhong, Hui Wang, Ningping Tao

**Affiliations:** ^1^College of Food Science and Technology, Shanghai Ocean University, Shanghai, China; ^2^School of Public Health, Shanghai Jiao Tong University School of Medicine, Shanghai, China; ^3^Henan Commerce Science Institute Co., Ltd., Zhengzhou, China; ^4^Xinhua Hospital, Shanghai Institute for Pediatric Research, Shanghai Key Laboratory of Pediatric Gastroenterology and Nutrition, Shanghai Jiao Tong University School of Medicine, Shanghai, China

**Keywords:** apigenin, emulsion stability, *in vitro* digestion, lipolysis, bioaccessibility

## Abstract

Few studies have investigated the encapsulation of apigenin in solid particle-stabilized emulsions. In this work, Pickering emulsions containing apigenin and stabilized by whey protein isolate-chitosan (WPI-CS) complexes were created to enhance the bioavailability of apigenin. Different lipids including medium-chain triglycerides (MCTs), ethyl oleate (EO), and corn oil (CO) were selected to fabricate lipid-based delivery systems. The microstructure of the Pickering emulsions, as revealed by optical and cryo-scanning electron microscopies, showed that the oil droplets were dispersed evenly and trapped by a three-dimensional network formed by the WPI-CS complexes, which was further confirmed by rheology properties. After 30 days of storage, Pickering emulsions with MCTs achieved the highest apigenin retention rate, exhibiting 95.05 ± 1.45% retention when stored under 4°C. *In vitro* gastrointestinal tract experiments indicated that the lipid types of the emulsions also affected the lipid digestion and release rate of apigenin. Pickering emulsions with MCTs achieved a higher bioaccessibility compared to that of the other two emulsions (*p* < 0.01). These results indicate that the delivery system of Pickering emulsions with MCTs stabilized by WPI-CS complexes offers good storage stability and improved bioaccessibility of apigenin.

## Introduction

Apigenin, 4',5,7-trihydroxyflavone, is a flavonoid, particularly abundant in the ligulate flowers of the chamomile plant (1), and also found in other sources such as celery (2), parsley (3), peppermint, and grapefruit (4). In recent years, apigenin has attracted great interest in the food and pharmaceutical industries due to its biological activities (5). Accumulated evidence revealed that apigenin possesses many pharmacological activities, including antioxidant (6), anti-inflammatory (7), anti-carcinogenic (8), and neuroprotective (9, 10). However, the low solubility and poor bioavailability in the human body restrict the clinical use of apigenin (11).

Encapsulation technology can improve the solubility or stability of bioactive substances in water as well as oral bioavailability. The emulsion delivery system is one of the most widely used encapsulation methods in the food and pharmaceutical industry. To date, several studies have reported encapsulation of apigenin in emulsions using small-molecular-weight surfactants or biopolymers acting as emulsifiers or stabilizers. For example, Abcha et al. reported oil(O)/water(W) submicron emulsions stabilized by Tween 20 for encapsulating apigenin (5), Zhao & Wang constructed apigenin-loaded O/W microemulsions using Tween 40 and S1570 as the surfactant phase (12), and Kim et al. developed multiple apigenin emulsions stabilized by Tween 80 (13). However, small molecular surfactants present toxic effects when they are added in large amounts (14). Moreover, few developments in solid particle-stabilized emulsions for encapsulating apigenin were reported so far.

The solid-particle-stabilized emulsions are generally known as Pickering emulsions. Compared to conventional emulsions, they have the advantages of biocompatibility, better stability against coalescence and Ostwald ripening (15, 16), and higher bioaccessibility as a carrier for transporting bioactive substances (17). Consequently, Pickering emulsions show great potential for applications in the food and pharmaceutical industry (18–20). Proteins, polysaccharides, and their complexes are often used as Pickering stabilizers in the encapsulation processes (21–23). However, Pickering emulsions with compound emulsifiers show many advantages over those with protein or polysaccharide alone.

Electrostatic interactions or covalent bonds are considered the possible mechanisms by which proteins and polysaccharides interact to form supramolecular complexes (24). Steric hindrance and electrostatic interactions between proteins and polysaccharides help to form a special three-dimensional (3D)-network structure between droplets (25), which enhances the stability of the emulsion. Furthermore, polysaccharides have a hydrophilic chain segment extending from the surface, which enhances spatial stability and binds to a large amount of water, thereby promoting the stability of the emulsion (25). Apart from the hydrophobic groups present at the O/W interface, the proteins exhibit a large number of hydrophilic peptides, which extend from the oil droplet surface and increase the thickness of the stable layer, further enhancing the emulsion stability (26). In addition, the presence of proteins and polysaccharides increases the possibility of binding between the active substances and particles, which endows the emulsion with better loading properties (27). In terms of proteins, whey protein isolates (WPI) have been identified as promising stabilizers.

WPI contains large amounts of the functional protein β-lactoglobulin, a small amount of α-lactalbumin, and bovine serum albumin (28). Chitosan (CS), obtained by N-deacetylation of chitin, is well known as a naturally occurring cationic polysaccharide with remarkable properties such as biocompatibility, biodegradation, low immunogenicity, and non-cytotoxicity (29–31). WPI interacting with CS can form soluble or insoluble complexes. A previous study reported that nanocomplexes derived from WPI and CS were used to encapsulate bioactive substances such as anthocyanins and omega-3 fatty acids, showing good stability or slow-release effect (32, 33).

The use of nanoemulsions as carriers of hydrophobic compounds was shown to improve bioaccessibility (34). The ability of lipids and hydrophobic compounds to form mixed micelles is the key to the absorption of many hydrophobic compounds (35). This is also highly correlated with the digestibility of oil droplets constituting the emulsions (36). Furthermore, the higher the concentration of free fatty acids (FFAs) produced during lipid digestion, the higher is the concentration fraction of lipids in micelles, and the stronger is the ability of hydrophobic compounds to form micelles and increase bioaccessibility (37). This relationship may be affected by the emulsion properties and lipid composition including fatty acid chain length (38) and degree of unsaturation (39). Therefore, the lipid type has a significant impact on bioaccessibility.

In this study, medium-chain triglycerides (MCTs), ethyl oleate (EO), and corn oil (CO) were selected as oil-phase solvents for apigenin. The WPI-CS complexes were used as emulsifiers and stabilizers to construct Pickering emulsions that improve the bioavailability of apigenin. The morphological characteristics of the obtained emulsions were determined by a variety of microscopic techniques, and the rheological properties were also investigated. The storage stability of the Pickering emulsions was evaluated and compared by analyzing the particle size and retention rate of apigenin under different temperatures. The physical properties and digestibility of apigenin encapsulated in the Pickering emulsions were studied during a simulated gastrointestinal tract (GIT). The influence of lipids on the *in vitro* lipid digestibility kinetics and their relationship with the apigenin bioaccessibility were also evaluated. The findings obtained in this work provide a reference for building solid particle-stabilized delivery emulsions to enhance the bioavailability of apigenin.

## Materials and methods

### Materials

Chitosan was obtained from Tokyo Chemical Industry, while WPI and MCTs were provided by Shanghai Yuanye Bio-Technology Co., Ltd. EO was purchased from J&K Scientific Ltd. CO was purchased from Innochem Co., Ltd. Apigenin, glycerol, and mucin from the porcine stomach, pepsin from porcine gastric mucosa (250 units/mg solid), bile salts and lipase from porcine pancreas (100–650 units/mg protein) were purchased from Sigma-Aldrich (Sigma Chemical Co. St. USA). All the solvents and reagents were of analytical grade. The double-distilled water used in this study was obtained from a water purification system (Direct-Pure-EDI 15UV).

### Preparation of CS solution

The CS solution was prepared using a method described by Wang et al. (18), with minor modifications. Briefly, 1.5% (w/v) stock CS solutions were prepared by dissolving 7.5 g CS powder into 500 mL of 1.0% (v/v) aqueous acetic acid solution and stirring at 700 rpm for 2–4 h. The solution was then left to stand for 12 h to allow complete dissolution and hydration.

### Preparation of WPI solution

Twenty g of WPI were dissolved in 200 mL of ultrapure water and fully mixed. Then, the solution was placed in a shaking water bath (SWT-100; MIU Lab, China) and kept at 90°C for 30 min. Finally, the WPI solution was placed in the refrigerator at 4°C overnight, for further use.

### Preparation of emulsion

**Figure 1:**
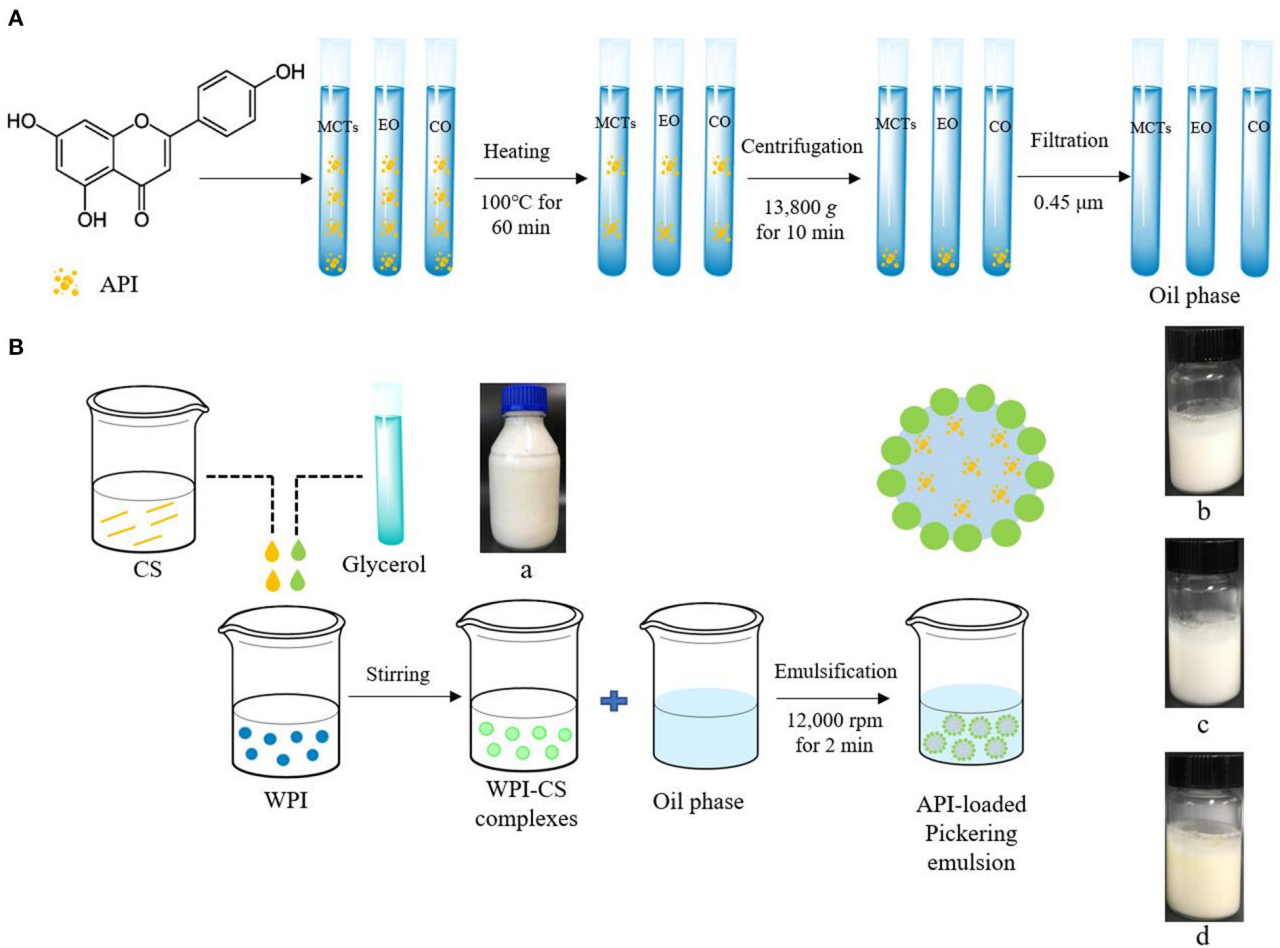
Schematic diagram of preparation procedure of apigenin (API)-loaded Pickering emulsion with MCTs (b), EO (c), and CO (d) stabilized by WPI-CS complexes (a). **(A)** indicates the preparation procedure of oil phase; **(B)** indicates the preparation procedure of API-loaded Pickering emulsion.

The schematic diagram of the preparation procedure of the apigenin-loaded Pickering emulsion is shown in Figure 1.

#### Preparation of oil phase

The oil phase containing dissolved apigenin was prepared according to Abcha et al. (5), with minor changes. First, 0.15 g of apigenin were dissolved in 100 mL of each MCTs, EO, and CO by stirring at 500 rpm for 30 min using a magnetic stirrer (RH basic 1, IKA, Germany). Thereafter, the samples were heated in a water bath at 100°C for 60 min, followed by stirring overnight at 500 rpm at room temperature. The next day, the solutions were centrifuged at 13,800 *g* by a high-speed centrifuge (H2050R; Xiangyi Instrument Co., Ltd., Changsha, China) for 10 min. After centrifugation, the sediment was removed, and the supernatant was filtered through a 0.45-μm filter membrane. The concentration of apigenin in the final oil phase with MCTs, EO, and CO were 0.96 ± 0.03, 0.60 ± 0.01, and 0.87 ± 0.02 mg/mL respectively.

#### Preparation of WPI-CS complexes

The CS stock solution was induced by adjusting the pH to 6.4–7.0 using 1.0 M NaOH. The 1.5% CS solution was then mixed with 10% WPI solution, in the same proportion, and glycerol was added as a crosslinking agent at 2% (w/v). Following this procedure, a complex solution containing 0.75% CS and 5% WPI was obtained (32).

#### Preparation of apigenin-loaded Pickering emulsion

To obtain apigenin-loaded picking emulsion, 7 mL of the oil phase and 3 mL of the WPI-CS complexes were added into a 20 mL transparent glass bottle and then emulsified with a high-speed homogenizer (T18, IKA, Germany) at 12,000 rpm, for 2 min.

### Scanning electron microscopy

Samples of CS and WPI solutions and WPI-CS complexes, which were prepared as described in the preceding section, were freeze-dried using a SCIENTZ-10ND lyophilizer (Ningbo Scientz Biotechnology Co., Ltd., China). Then, the morphologies of the samples were investigated by SEM (HITACHI, Regulus 8100, Japan). Before visualization, these samples were sprayed with gold for 20 s, to make them conductive.

### Atomic force microscopy

Samples used for AFM analysis, including CS, WPI, and WPI-CS complexes, were prepared as described in the preceding section. AFM was performed using a Nanoscope V Multimode 8 (Bruker Corporation, USA). Data was analyzed using the NanoScope analysis software.

### Rheological properties

We investigated the rheological behavior of apigenin-loaded emulsion samples in terms of the apparent viscosity (η), storage modulus (*G*'), and loss modulus (*G*″) using a rheometer (MCR302, Anton-Paar, Austria). The detection methods used in this study were similar to those used in previous studies (18, 40).

### Measurement of droplet size and zeta (ζ) potential

The average droplet size of the Pickering emulsion samples in the storage experiment and the ζ potential of the samples collected from the GIT experiment were measured using a Malvern Particle Meter (NanoZS90; Malvern Instruments Ltd., UK). Before determination, the samples were diluted with phosphate buffer solution (PBS). The analyses were repeated three times to obtain average data.

### Optical microscopy

The sample microstructures were assessed using an OLYMPUS BX 53 microscope (Japan) equipped with an OLYMPUS DP74 camera. A 5 μL Pickering emulsion sample was placed on a glass slide, covered gently with a cover glass, and then placed on the microscope for observation.

### Cryo-scanning electron microscopy

The microstructures of the droplets in the apigenin-loaded Pickering emulsions were investigated using a cryo-SEM (SU8010; HITACHI, Japan) equipped with Everhart–Thornley and solid-state detectors. Before visualization, the samples were quickly frozen in nitrogen (−140°C) and sputtered with gold.

### Confocal laser scanning microscopy

The microstructures of all samples at different stages of the GIT experiment, were determined by CLSM (TCS SP8; Leica, Germany). The samples were dyed with fluorescein isothiocyanate (FITC) and Nile red before visualization. The FITC solution was prepared using dimethyl sulfoxide (DMSO) at a concentration of 10 mg/mL, and Nile red was dissolved in ethanol at a concentration of 1 mg/mL. Prior to observation, a 2 mL sample was poured into a 4 mL Eppendorf (EP) tube. Then, 100 μL of each FITC and Nile red solution were added successively for dying, stirred for 2 min using a vortex oscillator (Vortex-Genie2, Scientific Industries, USA), and placed in the dark for 5 min. For observation, 5 μL of the stained sample was placed on a slide, gently covered with a cover glass, and then subjected to CLSM. The obtained images were examined using the CLSM image analysis software (Leica Application Suite X, Leica, Germany). The excitation wavelengths of FITC and Nile red are 488 and 543 nm, respectively, while their emission wavelengths are 515 and 605 nm, respectively.

### Measurement of fatty acid content

Fatty acids of MCTs, EO, and CO were analyzed using gas-chromatography (Agilent 7890b, Agilent Co., USA) according to the China National Standards method of GB 5009.168-2016 (Commission, 2016). The analyses were repeated two times to obtain average data.

### Measurement of apigenin concentration

Samples of the Pickering emulsions containing apigenin were stored at temperatures of 4, 25, and 40°C avoiding light for 30 days. To evaluate the retention rate of apigenin in the Pickering emulsions during the stability experiments, the apigenin concentration in samples stored at different days were analyzed. To evaluate the release rate of apigenin during the GIT experiments, the apigenin concentration of the samples collected from the simulated gastric fluid (SGF) and simulated intestinal fluid (SIF) at set intervals were analyzed. The testing followed the procedure described by Zhang et al. (35) and Zhao et al. (41), with some modifications.

For a typical stability testing experiment, 200 μL samples of the emulsion in parallel for each group were added to 1 mL of ethanol. The obtained solution was mixed for 2 min using a vortex oscillator, then centrifuged at 18,407 *g* (Eppendorf 5424, Germany) for 10 min at 4°C. Then, the supernatant was removed, and its absorbance was measured at 340 nm using a Synergy H1 spectrophotometer (BioTek, USA). The retention rate was calculated by comparing the apigenin concentrations in the stored samples to those in the original emulsion samples.

For a typical testing procedure of the release rate of apigenin during *in vitro* simulation experiments, 0.5 mL samples were taken in parallel for each group every 20 min during SGF and SIF experiments and placed in a refrigerator at 4°C. After the GIT experiments, the collected samples were centrifuged at 18,407 *g* for 60 min at 4°C. Then, the supernatant was collected, its volume was recorded, and ethanol was added at an appropriate proportion. The other testing procedures followed the aforementioned procedure.

The release rate of apigenin was calculated based on the apigenin concentration in the hydrolysate solution collected from the GIT experiments. Then, the results were compared to those in the original emulsion samples.

### *In vitro* digestion model

In this experiment, the GIT model included simulated mouth, stomach, and intestine fluids. The simulated digestive fluids were prepared according to previous researches (35, 42), with minor modifications, as follows.

Initial apigenin-loaded emulsion samples were prepared as described in the preceding section. For GIT experiment, 5 mL of apigenin loaded emulsion and 15 mL of PBS were mixed and placed into an incubated shaker (Shaking water bath, SWT-100; MIULab, China) at 37°C for 30 min.

Simulated oral stage: 0.6 g mucus protein was added into 20 mL simulated saliva fluid (SSF), then fully mixed and placed in a water bath at 37°C for 30 min. After mixing with the initial emulsion, the pH was adjusted to 7.0 with 1 mol/L NaOH solution, and then the mixture was placed in a water bath shaker (37°C) and vibrated at 150 rpm for 10 min.

Simulated stomach stage: 0.064 g pepsin was added to 20 mL simulated stomach solution. The mixture was preheated in a water bath at 37°C for 30 min, then mixed evenly with 20 mL reaction solution from the oral stage. The pH of the mixture was adjusted to 2.5 with 1 mol/L HCl, and then placed in a water bath shaker (37°C) and oscillated at 150 rpm for 2 h.

Simulated small intestine stage: 20 mL simulated small intestine liquid was poured into a 100 mL graduated test tube containing 0.28 g bile salts and preheated in a water bath at 37°C for 30 min, followed by mixing with 30 mL reaction solution from the simulated stomach stage. Then the mixed solution was transferred into the reaction vessel of the full-automatic potentiometric titrator (Metrohm, 905 USA Inc.), the pH was adjusted to around 7.0, 3.0 mL of lipase (10 mg/mL) was added to the reaction solution, then the monitoring system of the full-automatic Potentiometric titrator was started immediately. During the 2 h reaction process (37°C), the reaction solution was titrated with 0.10 mol/L NaOH to maintain the reaction system pH at 7.0, and the volume of NaOH solution consumed was recorded. The GIT experiments were repeated two times.

### Release rate of FFAs

The release rate of FFAs was calculated by referring to the work reported by Li and McClements (43) and Zhang et al. (35) as:


$$FFA\;\left(\%\right)=\frac{\;C_{NaOH}\;•V_{lipid}\;•M_{w,lipid}}{2W_{lipid}}\;\times \;100,$$

where *C*_NaOH_ represents the concentration of NaOH titrant (in mol/L), *V*_lipid_ is the volume of NaOH solution consumed in the reaction, recorded by the automatic potentiometric titrator (in mL), *M*_w, lipid_ means the average molecular weight of MCTs, EO, and CO (g/mol), and *W*_lipid_ is the total mass of grease (MCT_S_, EO, and CO) in the emulsion (g).

### *In vitro* bioaccessibility

*In vitro* bioaccessibility experiments were performed as described in previous studies (17, 38, 44), with some modifications. After the simulated GIT experiment, the reaction solution was collected, and the total volume was recorded. Then, the solution was ultracentrifugated (Optima XPN-100, Beckman, USA) (186,000 *g*) for 1 h at 4°C, the supernatant was collected, and the bioaccessibility percentage of apigenin in the supernatant was calculated as:


$$\begin{array}{l} Bioaccessibility\;\left(\%\right)\\ =\frac{amount\;of\;solubilized\;apigenin\;in\;micelle}{amount\;of\;apigenin\;in\;original\;emulsion}\times \;100. \end{array}$$

### Statistical analysis

The Origin Pro 2018 software (OriginLab Corporation, USA) was used for data processing and mapping of the rheological property results. The retention rate of apigenin in the storage experiment, release kinetics of apigenin, bioavailability of apigenin in the GIT experiment, and particle size and ζ potential were processed with GraphPad Prism 7.0 software (GraphPad Software, USA). The released rate of FFA was processed using MS Excel 2019 software (Microsoft Inc., USA) and plotted by GraphPad Prism 7.0 software. The results were expressed as mean ± standard deviation (SD).

## Results and discussion

### SEM of WPI-CS complexes

**Figure 2:**
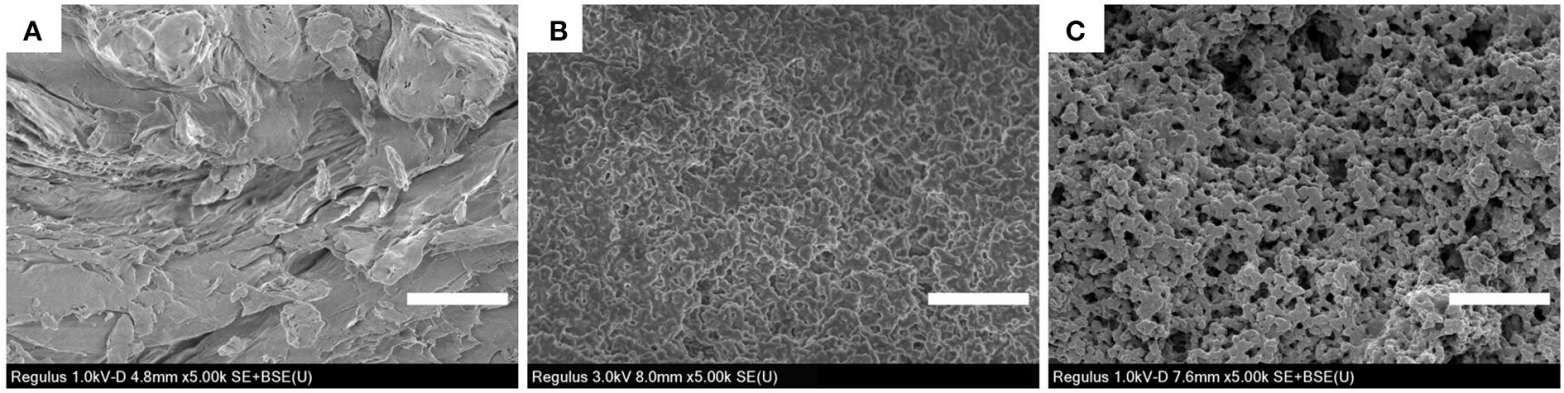
SEM of CS **(A)**, WPI **(B)**, and WPI-CS complexes **(C)**. Scale bars correspond to 5 μm.

As shown in Figure 2, the morphological characteristics of CS, denatured WPI, and WPI-CS complexes were significantly different. The morphology of CS was flat and dense; the morphology of denatured WPI was relatively uniform, stereoscopic, and dense; while the WPI-CS complexes presented an even porous network structure with uniform pores, which resulted from the interaction of CS and WPI with oppositely charged macromolecules, thereby generating a complex condensation network (21). As summarized in the introduction, the special structure of the WPI-CS complexes can enhance spatial stability and bind to a large amount of water, thereby promoting the stability of the emulsion.

### AFM of WPI-CS complexes

**Figure 3:**
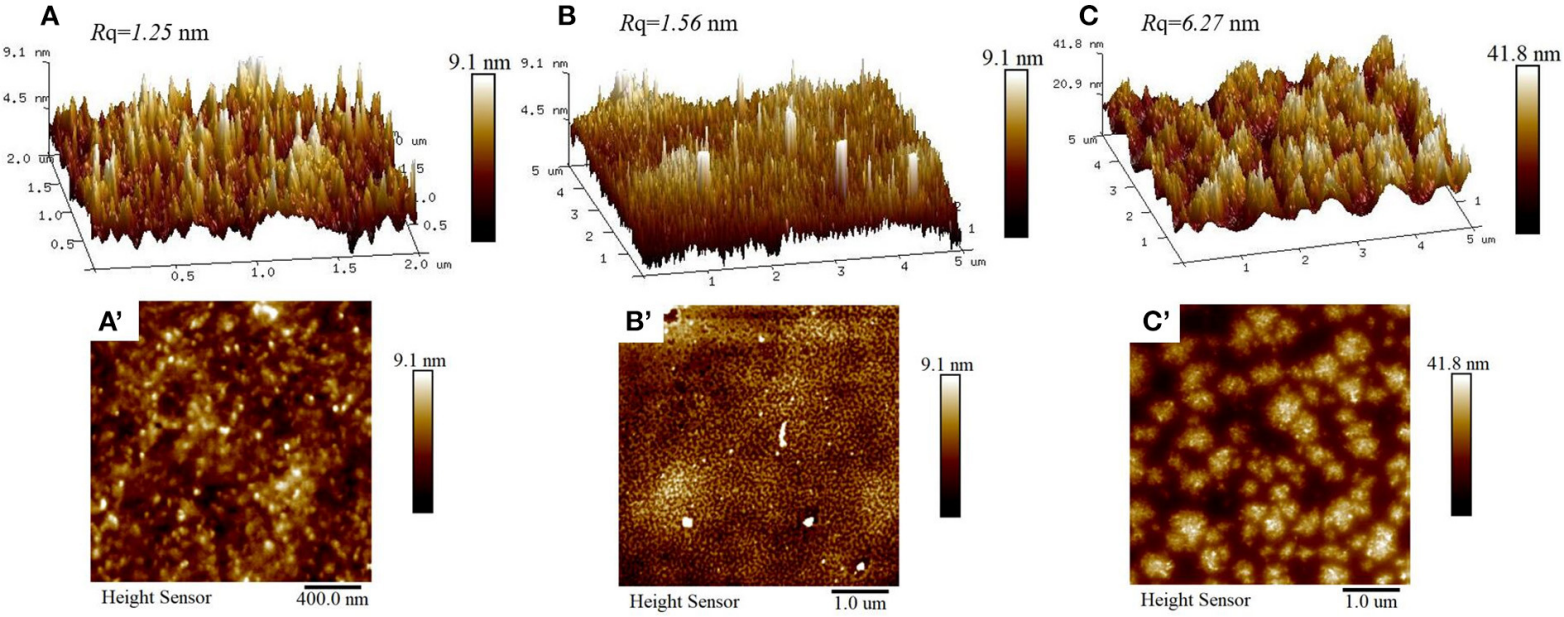
AFM 3-D (top) and height (bottom) images of CS **(A,A′)**, WPI **(B,B′)**, and WPI-CS complexes **(C,C′)**.

The adsorption morphologies of CS, WPI, and WPI-CS complexes were studied by AFM (Figure 3). The AFM images indicated that the surface of CS was fluctuant and exhibited a “peak” in the surface roughness of only 1.25 nm, while the height of CS ranged from 4.5 to 9.0 nm. Compared to CS, the surface of WPI was also fluctuant, although the shape of the “peak” seemed “sharper”, exhibiting a surface roughness of 1.56 nm, while the height of WPI ranged from 4.5 to 9.0 nm (i.e., the same as CS). For WPI-CS complexes, the droplet size and aggregation morphology changed significantly, forming a large aggregate with a surface roughness of 6.27 nm. The contour size was increased, with the particle height of 20–40 nm. The increase in droplet size means that the desorption energy increases correspondingly after the particle is adsorbed on the O/W interface. According to the Pickering stability mechanism (45), the stability of the emulsion stabilized by the WPI-CS complexes can increase in comparison to the emulsion stabilized by CS or WPI separately.

### Morphological characterization of apigenin-loaded Pickering emulsion

**Figure 4:**
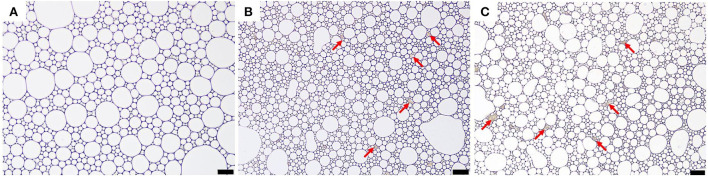
Optical micrographs of apigenin-loaded emulsions stabilized by WPI-CS complexes with MCTs **(A)**, EO **(B)**, and CO **(C)**. Scale bars of optical micrographs correspond to 50 μm.

**Figure 5:**
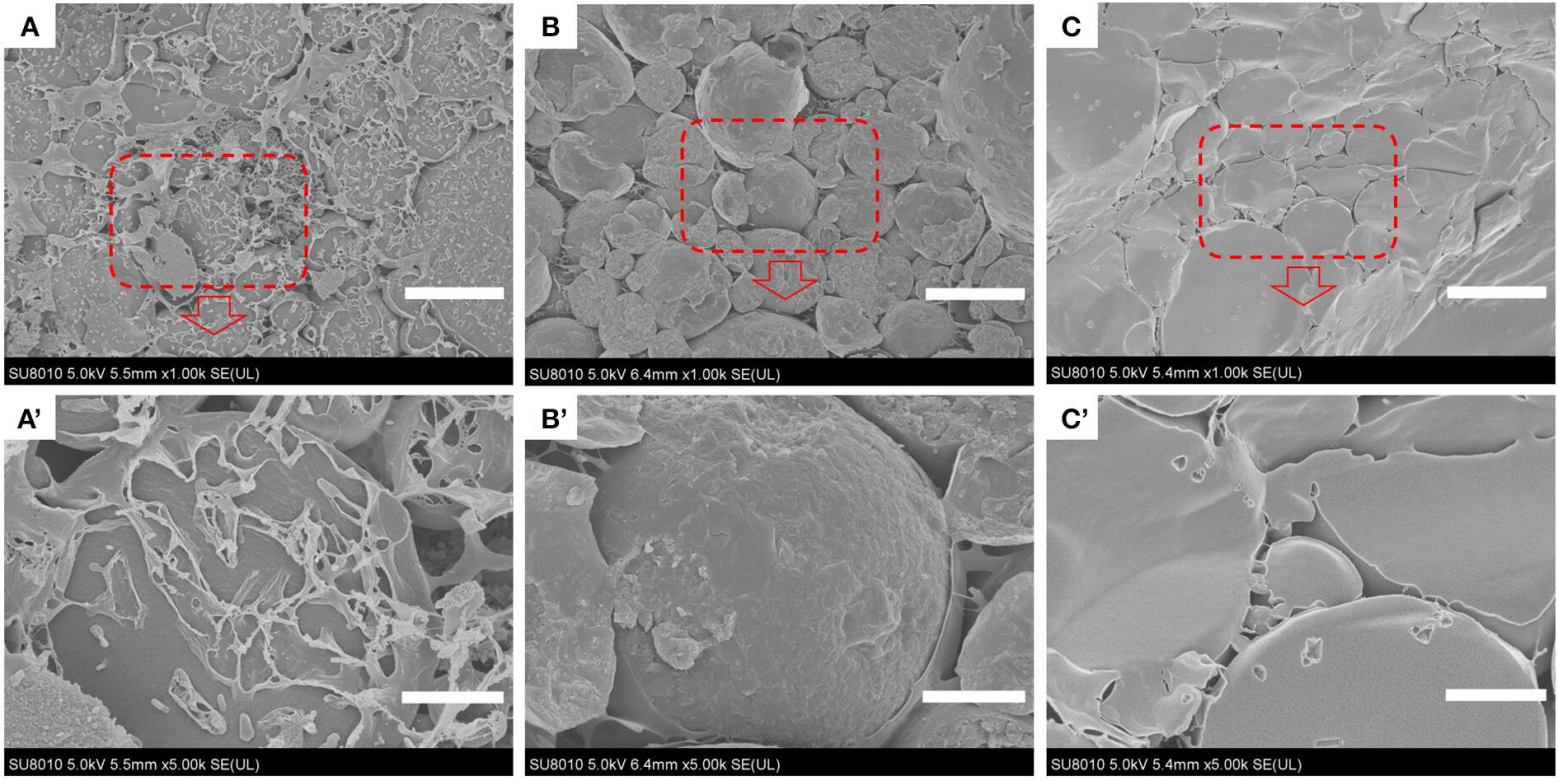
Cryo-SEM images of apigenin-loaded emulsions stabilized by MCTs **(A,A′)**, EO **(B,B′)**, and CO **(C,C′)**. Scale bars correspond to 25 (top) and 5 μm (bottom), respectively.

Analysis of the microstructure of emulsions (such as the interface structure, particle distribution, continuous phase network structure, etc.) helps understand the formation and stability of emulsions. Optical micrographs and cryo-SEM images of the apigenin-loaded emulsion samples are shown in Figures 4, 5, respectively. It can be intuitively seen from the optical micrographs that the oil droplets of the Pickering emulsions wrapped by WPI-CS complexes dispersed without aggregation, and that the oil droplets were trapped by a 3D network structure formed by WPI-CS complexes as shown in Figure 5. The 3D structure of the emulsions formed with MCTs emphasized the steric hindrance against the coalescence of droplets. From the optical images, variations in the distribution of oil droplets in the emulsions with different lipids are apparent. As shown in Figure 4, the droplets of the emulsions with MCTs were distributed compactly and uniformly, exhibiting no visible spaces between them, whereas for the other two types (emulsions formed with EO or CO), some evident spaces between the oil droplets were visible.

### Rheological characterization of apigenin encapsulated Pickering emulsions

**Figure 6:**
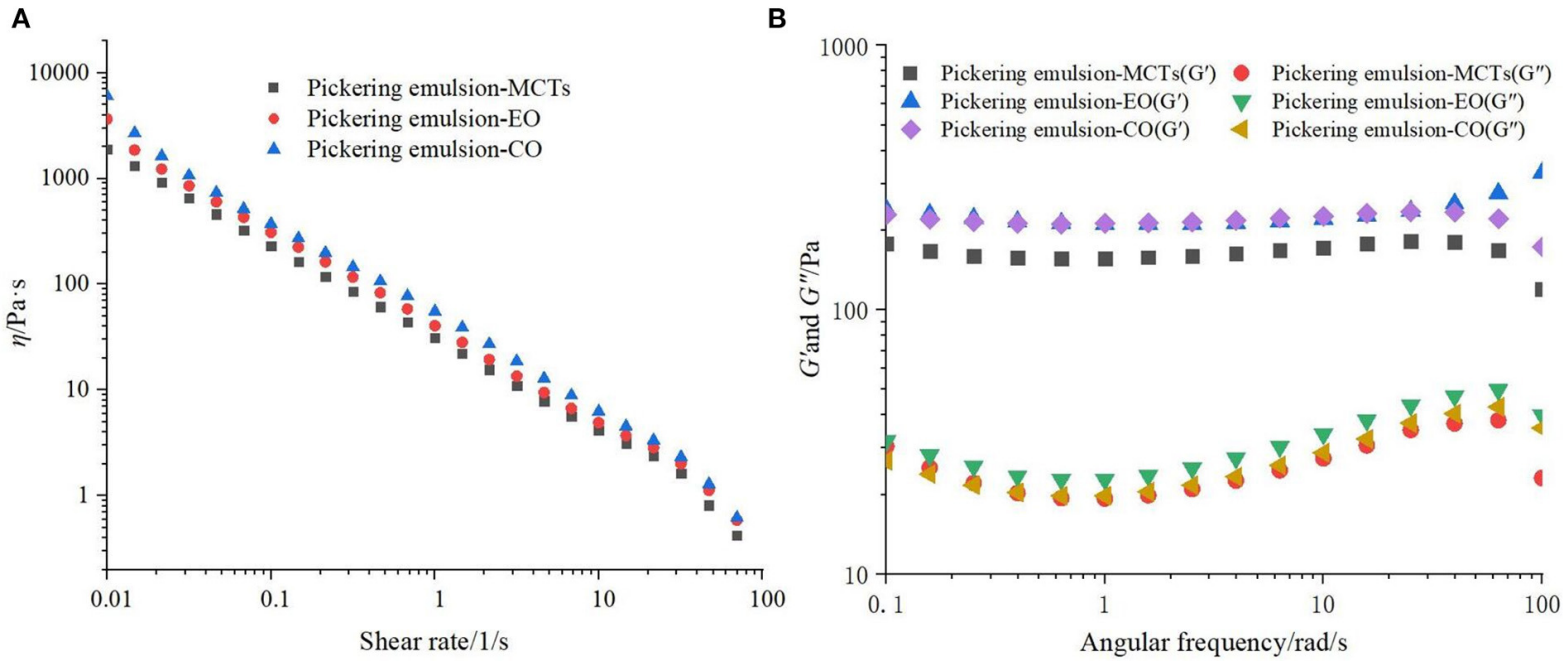
**(A)** Rheological characterization and **(B)** storage modulus (*G*′) and loss modulus (*G*″) of apigenin-loaded Pickering emulsions with different lipids (MCTs, EO, and CO).

A rheological characterization can evaluate whether an emulsion is stable and reveal the stabilization mechanism. The apparent viscosities (η) of the apigenin Pickering emulsions loaded with different oils (MCTs, EO, and CO) were similar, as shown in Figure 6A. All emulsions presented a shear-thinning behavior, which is characteristic of emulsions of pseudoplastic fluids in non-Newtonian fluids (44). Over the entire frequency range, the storage modulus (*G*′) in all Pickering emulsions was much larger than the corresponding loss modulus (*G*″), as depicted in Figure 6B. This result suggested that the emulsions were predominantly elastic, exhibited a 3D network structure, and the droplets presented a high degree of reversible deformation (46). The 3D network was mainly attributed to the closed packing of the oil droplets and bridges formed between them in the apigenin-loaded Pickering emulsions. This finding correlated well with the cryo-SEM results.

### Stability of apigenin in Pickering emulsions

**Figure 7:**
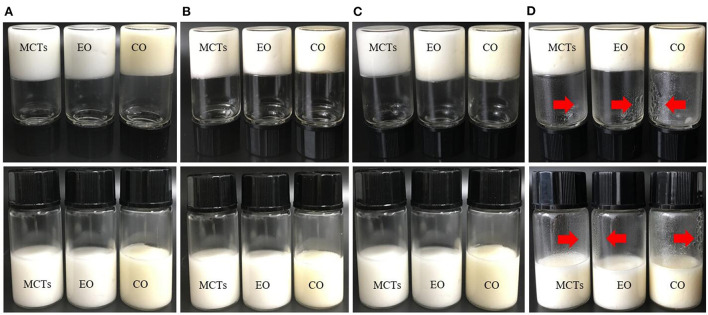
Visual observation of Pickering emulsions formed with different oils (MCTs, EO, and CO) stored under 4°C **(B)**, 25°C **(C)**, and 40°C **(D)** for 30 days, compared to the freshly prepared samples **(A)**.

**Figure 8:**
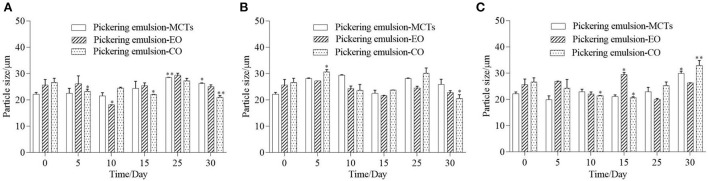
Droplet size of Pickering emulsions with different oils stored under 4°C **(A)**, 25°C **(B)**, and 40°C **(C)** for 30 days. “*” or “**” indicates a significant difference compared to the initial sample (*p* < 0.05, or *p* < 0.01).

**Figure 9:**
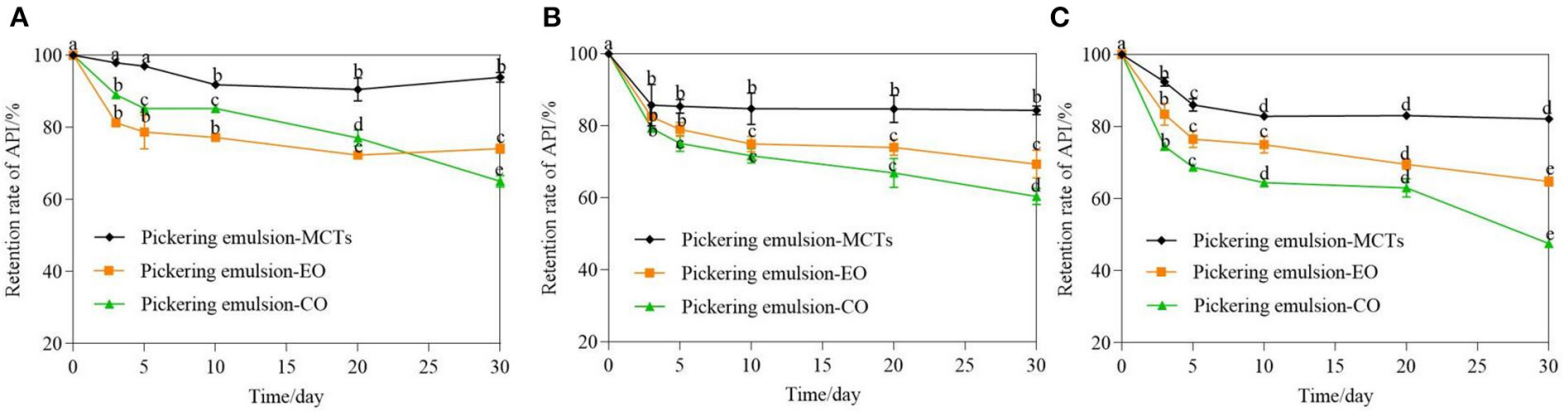
Retention rate of apigenin in Pickering emulsions with different oils (MCTs, EO, and CO) stored under 4°C **(A)**, 25°C **(B)**, and 40°C **(C)** for 30 days. a–e indicates the difference is significant (*p* < 0.05).

Storage temperature had an obvious effect on the Pickering emulsions, and Pickering emulsions may be unstable at high temperatures (40, 47). Therefore, it is necessary to investigate the effects of storage on the WPI-CS Pickering emulsions at different temperatures. Visual appearance, particle size, and retention rate of apigenin in the Pickering emulsions under storage temperatures of 4, 25, and 40°C were evaluated, and the results are presented in Figures 7–**9**, respectively.

Figure 7 shows that the color of the Pickering emulsions created with MCTs and EO is milk-white, which is caused by the colorless MCTs and EO, while the Pickering emulsions formed with CO exhibit a light-yellow color because of the yellow CO color. After 30 days of storage, the three samples stored at 4, 25, and 40°C exhibited no obvious appearance changes and no visible phase separation under the experimental temperatures. However, some water droplets were observed on the walls of the sample bottles stored at 40°C (Figure 7D), which was probably caused by partial evaporation of water in the emulsion at high temperatures.

The droplet size of the Pickering emulsion samples stored under different temperatures was measured as shown in Figure 8. The initial average droplet sizes of the emulsions formed with MCTs, EO, and CO were 22.1 ± 6.8, 25.6 ± 9.5, and 26.6 ± 7.3 μm, respectively, which was similar to the droplet size of Pickering emulsion stabilized by soy protein isolate-chitosan nanoparticles which ranged from 11.80 ± 2.20 to 43.68 ± 4.12 μm (40). After being stored for 30 days at 4°C, the droplet size of the Pickering emulsion samples exhibited fluctuations and a slight increase in the average value compared to the freshly prepared samples. The average droplet size of the Pickering emulsions with MCTs, EO, and CO fluctuated between the initial droplet size and the highest value of 28.5 ± 1.4, 29.3 ± 8.3 and 27.2 ± 9.6 μm, respectively. Furthermore, after being stored for 30 days at 25 and 40°C, the droplet sizes of the Pickering emulsions with MCTs and EO also fluctuated less than 30 μm, however, in this case, Pickering emulsions with CO fluctuated from their initial values to 30.6 ± 8.7 and 32.9 ± 9.5 μm, respectively. This increase in the droplet size and fluctuations during the higher-temperature storage indicated an aggregation of oil droplets that was probably caused by gravity separation (48). Previous research has also indicated a slight increase in droplet size in all submicron O/W emulsions stabilized by Tween 20 after 30 days of storage at 4°C (5).

The retention rate of apigenin in the Pickering emulsions with different lipids after storage at three different temperatures also showed a significant difference (Figure 9) (*p* < 0.01). After 30-day storage at 4°C, the retention rate of apigenin in the emulsion with MCTs was much higher than that in the other two samples during the whole storage period The retention rate of apigenin with EO decreased from 100% at day 0 to 72.28 ± 2.79% at day 20, and then changed slightly on day 30, while for the Pickering emulsions with CO, the retention rate of apigenin showed a downward trend during the whole storage period with the final retention rate of 65.06 ± 1.58% at 30 day. After storage at 25 and 40°C, the retention rate of apigenin in the Pickering emulsions with MCTs was also higher than that in the other two samples during the entire storage period. This was followed by the Pickering emulsions with EO. The apigenin retention in the Pickering emulsions with CO was lower than that in the other two samples, which was consistent with the results for droplet size (Figure 8). Meanwhile, the apigenin retention rates in Pickering emulsions formulated with the same oil phase also changed under different temperatures. For Pickering emulsions with MCTs stored at 4°C for 30 days, the apigenin retention rate changed little, with 95.05 ± 1.45% of apigenin retained in the emulsions, which was much higher than that at 25 and 40°C that exhibited 85.10 ± 1.20% and 82.14 ± 0.88% retention rates, respectively. Furthermore, the apigenin retention rate at 4°C was slightly higher than that obtained in a previous study in which approximately 91.5–93.5% of the original apigenin was retained in O/W emulsions stabilized by Tween 20 after 30 days of storage at 4°C (5).

Therefore, it can be concluded that all samples were less stable at high temperatures. This phenomenon could be explained by the temperature-dependent diffusion of the dispersed oil droplets through the continuous phase, which could result in droplet coalescence or flocculation and emulsion instability (49). Apart from the Brownian motion, the stability of the emulsions was probably also affected by the solubility of apigenin in the oils at different temperatures.

### Physical stability of apigenin-loaded Pickering emulsions during the GIT experiments

The physical stability of apigenin-loaded Pickering emulsions was evaluated by investigating the microstructure and electrical characteristics (ζ potential) at various stages of the GIT experiments.

**Figure 10:**
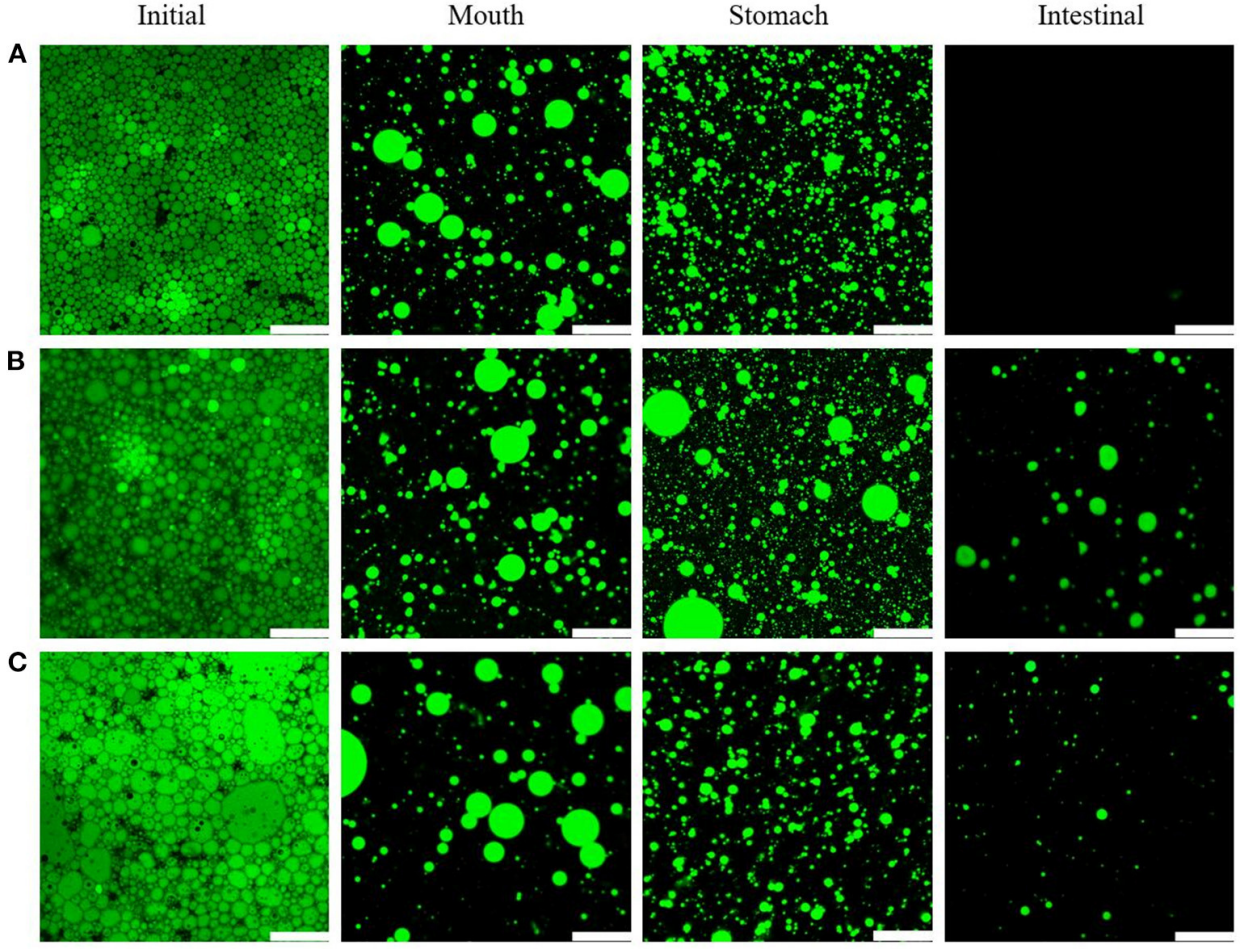
CLSM images of emulsions with different lipids MCTs **(A)**, EO **(B)**, and CO **(C)** exposed to different regions of simulated GIT. Scale bars correspond to 115 μm.

Figure 10 shows CLSM images of different lipid types in the apigenin-emulsions exposed to various stages of the simulated GIT. Initially, all the oil droplets (green color) appeared to be evenly distributed throughout the emulsions [Figure 10 (initial stage)]. However, the droplet size of the emulsions formed with MCTs was more uniform than that of the other two emulsions, which was consistent with the optical micrographs (Figure 4). The effect of the mucus protein in oral fluid on the emulsion at the oral stage was simulated. The CLSM image after the mouth stage showed that the oil phases in the three types of oral emulsions had different degrees of aggregation. The droplet aggregation may be caused by either bridging or depletion flocculation induced by the presence of mucin in the simulated saliva (50).

After the digestion by the simulated gastric fluid, the WPI wrapped on the surface of the lipid droplets was digested by pepsin (51). This explains the presence of large regions of uncovered interface which results in a certain amount of oil released. Figure 10 shows an increase in the number of oil droplets after gastric digestion compared to that during the mouth stage. Moreover, some droplet aggregation occurred (Figure 10).

In the intestinal phase, the main processes were lipid emulsification by bile salts and lipase hydrolysis by lipase. After the intestinal digestion phase, the CLSM images indicated significant differences in the emulsions obtained with the three oil types. No lipid droplets could be observed for the emulsions with MCTs, which indicated that MCTs had been hydrolyzed by lipase through the small intestine, while EO and CO emulsions contained indigested lipid droplets, which was consistent with the lipid hydrolysis results (see further).

**Figure 11:**
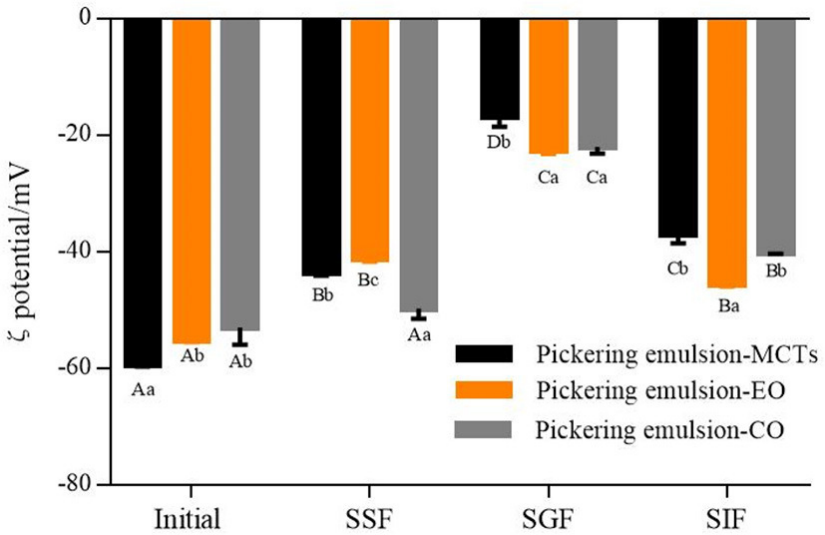
ζ potential of apigenin-loaded Pickering emulsions with different oils (MCTs, EO, and CO) exposed to different regions of simulated GIT. A–D indicates a significant difference for one sample at the different GIT phases (*p* < 0.05). a–c indicates a significant difference between different samples at the same GIT phase (*p* < 0.05).

The average ζ potential of apigenin-loaded Pickering emulsions formed with different oils (i.e., MCTs, EO, and CO) during the simulated GIT are shown in Figure 11. All the initial emulsions contained droplets of highly negative charge as quantified by zeta potential. The negative charge was mainly due to the presence of WPI-CS complexes on the surface of the droplets as shown in the microstructure. As discussed in the introduction, the electrostatic stabilization mechanism and steric hindrance work together to prevent droplet coalescence in emulsion systems (35). It is also generally regarded that particle charge is one of the factors governing the physical stability of emulsions: the higher the electrostatic repulsion between the particles, the higher is the physical stability (52). Notably, the emulsions with MCTs exhibited the highest ζ potential value of −59.35 ± 0.35 mV, which was followed by that of the emulsions with EO (−55.23 ± 0.28 mV) and CO (−53.03 ± 2.95 mV) (Figure 11). This was consistent with the stability results presented in the section “Stability of apigenin in Pickering emulsion”.

In the mouth phase, the ζ potential of the three samples showed a decreasing trend after the oral stage in comparison to the initial stage (Figure 11). The reason for this may be ascribed to the electrostatic shielding effect of the mineral ions in saliva, or the binding effect of mucin adsorption on lipid droplets that leads to the reduction of negative charge (51). Furthermore, the ζ potential of the particles in the emulsions with the different lipids also showed differences within the simulated mouth phase. The emulsions with MCTs and EO exhibited a lower ζ potential than those of the emulsions with CO.

In the gastric fluid, the hydrolysis of the proteins coated on the surface of the lipid droplets can result in the reduction of electrostatic repulsion (51, 53). Moreover, the low pH of the aqueous phase surrounding the oil droplets can result in a decrease in the negative charge (38). Combined, these effects resulted in the decline of the negative charge on the particles in all the emulsions made herein, as shown in Figure 11.

The composition of the simulated small intestine phase was complex, consisting of various types of colloidal particles, including pharmaceutical components released into the system, undigested protein aggregates, undigested lipid droplets, CS, FFAs, micelles, vesicles, and bile salts (54). It is also possible that the re-emulsification of oil was contributed to by the surface-active bile salts (55). Meanwhile, since most of the samples were anions, the ζ potential of the Pickering emulsion samples (ranging from −37.05 ± 1.48 to −45.7 ± 0.28 mV) during the small intestine stage exhibited a higher negative charge than the ζ potential of the samples during the stomach stage (which ranged from −16.8 ± 1.73 to −22.57 ± 0.66 mV). Previous research suggested that there was a relationship between the electrical charge characteristics and the lipolysis degree during *in vitro* digestion, and a lower ζ potential indicated a higher lipid digestion degree (56). However, in this study, the opposite results were obtained. Although the ζ potential of the emulsions with EO (−45.7 ± 0.28 mV) was significantly lower than those of emulsions with CO (−40.2 ± 0.1 mV) and MCTs (−37.05 ± 1.48 mV) (*p* < 0.05), as shown in Figure 11, lower lipid digestion of EO and CO was indicated from confocal images (Figure 10) and the lipid hydrolysis results. This may be caused by the small amounts of EO and CO residue remaining in the water phase due to the low hydrolysis rate, and the re-emulsification of undigested oil caused by the surface-active bile salts (55), both of which enhance the electrostatic repulsion of the particles.

During the GIT experiments, it seems that the emulsions with different lipids showed similar trends, however, the significant differences were observed in lipid digestion (to be further discussed) as indicated in the confocal microscopy images in Figure 10, there is no lipid in the emulsion with MCTs can be observed, while there is lipid can be seen from that of EO and CO based emulsion after *in vitro* digestion.

**Figure 12:**
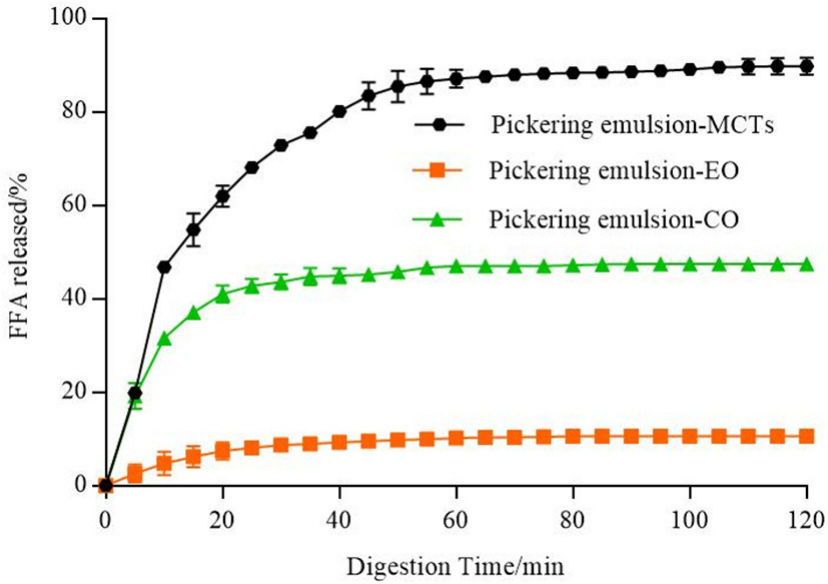
Amounts of fatty acids released from emulsions including different lipids (MCTs, EO, and CO).

*In vitro* lipid digestion of apigenin-loaded Pickering emulsions. The lipids used in emulsion delivery systems significantly influence the rate and extent of lipid digestion and release (57). As shown in Figure 12, the differences in the lipid hydrolysis of apigenin-encapsulated Pickering emulsions with different oils were obvious. FFAs released from the Pickering emulsions with MCTs were much higher than those from the other two emulsion samples during the 2 h of the simulated intestinal digestion, with the final release of FFAs from the MCT emulsions approaching 90%, followed by CO at approximately 50%. FFAs released from the emulsions with EO were the lowest (even lower than 20%), and the incomplete digestion of EO and CO (undigested droplets) can be seen in the confocal images in Figure 10.

Because of the differences in the water dispersity of FFAs formed by the digestion (54), the fatty acid composition of the lipids was analyzed as shown in Supplementary Table S1, with MCTs consisting of medium-chain fatty acids (MCFAs) with 54.71 ± 0.00% octanoic acid (C8:0) and 45.17 ± 0.00% decanoic acid (C10:0). EO consisted of 99.89 ± 0.00% LCFAs, including 75.27 ± 0.04 MUFAs, and 12.62 ± 0.02 PUFAs. CO consisted of 100.00 ± 0.00% LCFAs, including 27.28 ± 0.01% MUFAs, and 57.49 ± 0.00 PUFAs. As explained in previous reports (17, 58), the mid-chain fatty acid produced in the process of oil hydrolysis of MCTs has a high affinity for the water phase and can quickly migrate to the water phase without preventing the hydrolysis reaction on interfacial lipase. However, the long-chain fatty acids tend to accumulate at the O/W interface, reducing the ability of lipase to reach the lipid droplet to hydrolyze triglycerides, thus inhibiting the process of lipase hydrolysis. Therefore, the digestion rate of MCTs was much higher than that of the long-chain triacylglycerols (EO and CO), which confirmed the results of lipid digestion shown in Figure 12. Compared with long-chain fatty acid glycerides, MCTs have smaller molecular weights and faster hydrolysis rates, which makes them easier to be digested, absorbed, and metabolized by the human body (59).

### *In vitro* bioaccessibility of apigenin-loaded Pickering emulsions

**Figure 13:**
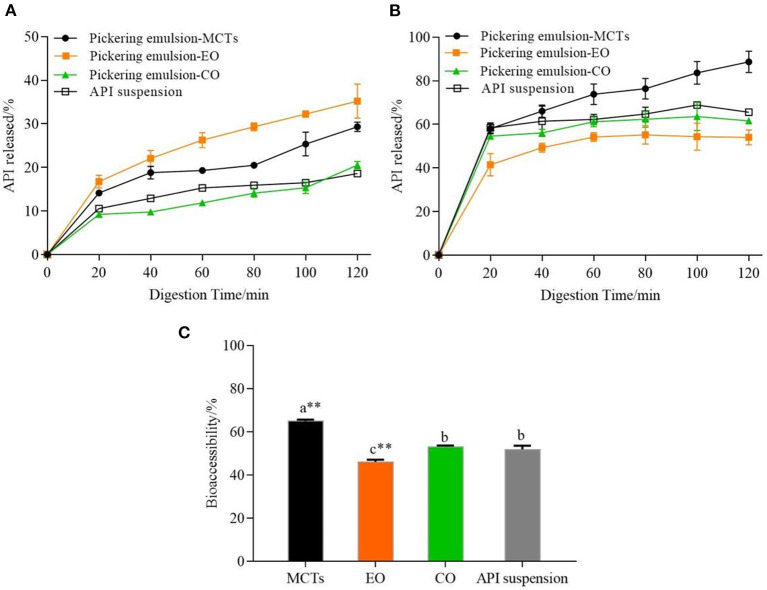
**(A)** Release kinetics of apigenin from Pickering emulsions during SGF stage, **(B)** Release kinetics of apigenin from Pickering emulsions during SIF stage, and **(C)** Comparison of bioaccessibility of apigenin between the Pickering emulsion samples. a–c indicates a significant difference between the different samples (*p* < 0.05). ** indicates that the difference is highly significant compared with apigenin (API) suspension (*p* < 0.01).

It was necessary to evaluate the stability of the Pickering emulsions during digestion based on the action mechanism of the oral agents in the human body (60). Figure 13A shows the release kinetics of apigenin from the Pickering emulsions under SGF, which is compared to the apigenin suspensions. Although increased during the 2 h of the simulated stomach digestion phase, the release kinetics of the apigenin from emulsions with different lipids showed significant differences. The apigenin release rate of emulsions with EO was the highest, followed by that of the emulsions with MCTs and CO, with the release rate of apigenin from CO emulsions being nearly equal to that of apigenin suspension. During the gastric digestion stage, the hydrolysis of proteins by pepsin occurs under the low pH (2.5) as mentioned above. Notably, it is generally regarded that Pickering emulsions are significantly demulsified by the enzymes under acidic conditions during gastric digestion (50). The release rates of apigenin may be related to the demulsification degree of emulsions with different oils in the simulated digestion stage, and also to the exposure of oil droplets to the water/oil interface (60).

Most nutrient absorption occurs during intestinal digestion, so it was desirable that the release of apigenin occurs during the intestinal digestion phase. As shown in Figure 13B, the apigenin release rates of the three emulsion samples in the small intestine were higher than those in the stomach digestion stage, which was as expected because of the sustained release effect of the Pickering emulsions on apigenin in the stomach. The release kinetics of apigenin from the Pickering emulsions with different lipids under SIF incubation also showed significant differences from those under SGF incubation. During the 2 h of the simulated intestinal digestion, the release rate of apigenin from the Pickering emulsions containing MCTs continued to increase and was consistently higher than those of the other samples, with the final release rate of 88.68 ± 4.91%. This value was higher than the values previously reported for apigenin released from apigenin-loaded polymeric micelles in an *in vitro* study (84%), however, the micelles were composed of Pluronic P123 and Solutol HS 15 (61). As the final apigenin release rate of emulsions containing CO was 61.56 ± 0.28%, which was higher than that of emulsions containing EO with 53.99 ± 3.38% (*p* < 0.01), however, it was still slightly lower than the apigenin suspension of 65.59 ± 1.48% (*p* < 0.01). Finally, the order of bioaccessibility of the Pickering emulsions prepared by different lipids shown in Figure 13C was as follows: MCTs (64.87 ± 0.75%), CO (51.63 ± 0.61%), and EO (48.41 ± 1.91%), which was consistent with the lipid hydrolysis rate as shown in Figure 12. It is generally considered that the amount of apigenin entering vesicles and micelles represents the bioaccessibility of apigenin after digestion (17). Compared with medium- chain FFAs (C8 and C10) in MCTs, a large number of long-chain unsaturated fatty acids in EO and CO, as analyzed in Supplementary Table S1, results in less lipid digestion products to form micelles, and therefore, low bioaccessibility. Meanwhile, the bioaccessibility of apigenin suspension was 51.33 ± 1.52%, which was lower than that of the Pickering emulsions with MCTs (*p* < 0.01), but higher than that of the Pickering emulsions with EO (*p* < 0.05), and not significantly different compared to the Pickering emulsions with CO (*p* < 0.05). Previous research has reported similar results. For example, the apigenin release rate of orange oil–Tween 80 emulsions was higher than that of the apigenin suspension; however other samples including soybean oil–Tween 80 emulsions, soybean oil–PGPR emulsions, and orange oil-PGPR emulsions exhibited lower release rates than apigenin suspensions (13). Overall, these findings indicate that both the stabilizers and lipid types have strong effects on the release rate and bioaccessibility of apigenin.

## Conclusion

In this paper, WPI-CS complexes were used to construct Pickering emulsions by embedding apigenin using different lipids. The microstructure and rheological characterization results showed that the obtained Pickering emulsions, constructed with an oil phase as the core, were dispersed uniformly and surrounded by a 3D network formed by WPI-CS complexes. After 30 days of storage under the test temperatures, apigenin encapsulated in the Pickering emulsion samples showed good sensory stability with no separation of oil and water. Notably, the highest apigenin retention rate of 95.05 ± 1.45% was attained by the Pickering emulsion with MCTs after 30 days of storage at 4°C, which was higher than that of the other two emulsions (with EO and CO). In the *in vitro* digestion experiments, the lipid type was found to have a significant influence on the lipolysis rate, apigenin release rate, and bioaccessibility, which was mainly caused by the different fatty acid chain lengths. In the gastric digestion phase, the release rate of apigenin in the EO oil phase was higher than that of the other two emulsions, however, in the intestinal digestion phase, the lipolysis rate, release rate, and final bioaccessibility of apigenin for Pickering emulsions with MCTs were higher than those of the other two emulsions, as expected. In summary, Pickering emulsions constructed with WPI-CS complexes formulated with MCTs as the oil-phase and embedding apigenin had better storage stability and higher bioaccessibility than the two emulsions created with EO and CO. Moreover, the delivery system was beneficial in improving the bioavailability of apigenin. Therefore, solid particle-stabilized emulsions can be used for encapsulating apigenin with the advantages including reducing toxic effects compared with small molecular surfactants. In future work, *in vivo* experiments will be conducted to further evaluate the digestive characteristics and bioaccessibility of the drug delivery system, as well as its functional properties.

## Data availability statement

The original contributions presented in the study are included in the article/Supplementary material, further inquiries can be directed to the corresponding author/s.

## Author contributions

RG, NT, HW, and JZ contributed to conception and design of the study. RG conducted the experiment of the study and wrote the first draft of the manuscript. RG and HZ performed the statistical analysis. JZ and HZ wrote sections of the manuscript. All authors contributed to manuscript revision, read, and approved the submitted version.

## Funding

This work was supported by Horizontal Project of Shanghai Jiao Tong University School of Medicine (2020HX005) and Provincial Distinguished Researcher Project of Henan Province (200511003).

## Conflict of interest

Author HZ was employed by Henan Commerce Science Institute Co., Ltd. The remaining authors declare that the research was conducted in the absence of any commercial or financial relationships that could be construed as a potential conflict of interest.

## Publisher's note

All claims expressed in this article are solely those of the authors and do not necessarily represent those of their affiliated organizations, or those of the publisher, the editors and the reviewers. Any product that may be evaluated in this article, or claim that may be made by its manufacturer, is not guaranteed or endorsed by the publisher.
