# Supplementary material for: Storage stability and in vitro digestion of apigenin encapsulated in Pickering emulsions stabilized by whey protein isolate–chitosan complexes
Source: Front Nutr. 2022 Sep 29;9:997706. doi: 10.3389/fnut.2022.997706 (PMC9556715; doi:10.3389/fnut.2022.997706)
Supplement: Supplementary file 1 [file Data_Sheet_1.PDF]

Supporting Information for

Storage Stability and *in vitro* Digestion of Apigenin Encapsulated in Pickering Emulsions Stabilized by Whey Protein Isolate–Chitosan Complexes

Table S1. Fatty acid composition of the lipids.

Fatty acid	MCTs/%	EO/%	CO/%
C8:0	54.71±0.00	-	-
C10:0	45.17±0.00	-	-
C12:0	-	0.11±0.00	-
C14:0	-	0.11 ±0.00	-
C16:0	-	8.02 ±0.01	12.69±0.00
C18:0	-	3.64 ±0.03	1.85±0.00
C18:1n9	-	74.86 ±0.03	27.06±0.01
C18:2n6	-	12.57 ±0.05	56.90±0.00
C18:3n3	-	0.11 ±0.00	0.59±0.00
C20:0	-	0.24 ±0.00	0.42±0.01
C20:1	-	0.41 ±0.01	0.22±0.01
C22:0	-	-	0.13±0.00
C24:0	-	-	0.15±0.00
MCFAs	100.00±0.00	0.11±0.00	0.00
LCFAs	0.00	99.89±0.00	100.00±0.00
SFAs	100.00±0.00	12.11±0.02	15.23±0.02
MUFAs	0.00	75.27±0.04	27.28±0.01
PUFAs	0.00	12.62±0.02	57.49±0.00

Medium-chain fatty acids (MCFAs) are fatty acids with carbon chain lengths of C6–C12; long-chain fatty acids (LCFAs) are fatty acids with carbon chain lengths of C14–C24; SFAs are saturated fatty acids; MUFAs are monounsaturated fatty acids; PUFAs are polyunsaturated fatty acids. The hyphen ("-") indicates that a value is below the detection limit.
